# Distribution of cervical abnormalities detected by visual inspection with acetic acid in Swaziland, 2011–2014: A retrospective study

**DOI:** 10.4102/phcfm.v10i1.1773

**Published:** 2018-10-18

**Authors:** Themba G. Ginindza, Maribel Almonte, Xolisile Dlamini, Ben Sartorius

**Affiliations:** 1 Discipline of Public Health Medicine, School of Nursing and Public Health, University of KwaZulu-Natal, South Africa; 2 Epidemiology Unit, Ministry of Health, Swaziland; 3 Prevention and Implementation Group, International Agency for Research on Cancer, Lyon, France

## Abstract

**Background:**

Cervical cancer is the fourth most common cancer worldwide among women, with the number of new cases increasing from 493 243 in 2002 to 527 000 in 2012. These numbers are likely to be underestimated because given the lack of registration resources, cervical cancer deaths are usually under-reported in low-income countries.

**Aim:**

To describe the distribution of and trends in visual inspection with acetic acid (VIA) to detected cervical abnormalities in Swaziland by reviewing records of VIA examinations performed at two main hospitals in Swaziland between 2011 and 2014.

**Setting:**

Mbabane Government Hospital and Realign Fitkin Memorial (RFM).

**Methods:**

Records of cervical screening using VIA at the Mbabane government hospital and RFM hospital between 2011 and 2014 were retrieved. Positivity rates (PRs) of VIA with 95% confidence intervals (95% CI) were calculated and used as proxies of cervical abnormalities. Odds ratios of the association between VIA-detected cervical abnormalities and human immunodeficiency virus (HIV) status were estimated using logistic regressions.

**Results:**

VIA was positive in 1828 of 12 151 VIA records used for analysis (15%, 95% CI: 14.4–15.7). VIA was positive in 9% (36 of 403) women under the age of 20, in 15.5% (1714 of 11 046) of women aged 20–49 years and in 11.1% (78 of 624) of women aged 50–64 years. A decreasing trend of VIA positivity was observed over time at both screening centres (*p* for trend < 0.001). Of 2697 records with Papanicolaou results, 20% (67 of 331) VIA-positives and only 5% (114 of 2366) VIA negatives had high-grade squamous intraepithelial lesion. Among 4578 women with reported HIV status, 1702 were HIV-positive (37.2%, 95% CI: 35.8–38.6). The prevalence of HIV in VIA-positive women was 62.5% (95% CI: 58.7–66.2), almost double that among VIA-negative women (33.0%, 95% CI: 31.6–34.5) and that among all women screened (*p* < 0.001). HIV-positive women were 3.4 times more likely to have cervical abnormalities on VIA than HIV-negative women (OR: 3.4, 95% CI: 2.8–4.0, *p* < 0.01).

**Conclusion:**

The high VIA PRs observed over four years in this study may reflect the prevalence of cervical abnormalities, in particular, in HIV-positive women. VIA is not a robust screening test, but it can play a major role in strengthening and expanding cervical cancer screening prevention programmes in resource-limited countries.

## Introduction

Cervical cancer is the fourth most common cancer worldwide among women, with the number of new cases increasing from 493 243 in 2002 to 527 000 in 2012, and the number of deaths slowly decreasing from 274 000 in 2002 to 265 000 in 2008.^1,2^ These numbers are likely to be underestimated because given the lack of registration resources, cervical cancer deaths are usually under-reported in low-income countries. Cervical cancer is likely the most frequent cancer type among women aged 15–45 years in Africa.^3^

Over 90% of all cervical cancer cases are caused by persistent infection with high-risk human papillomaviruses (hr-HPV),^3,4^ which can lead to pre-cancerous lesions and invasive cervical carcinoma (ICC) if not treated. However, cervical cancer is preventable at relatively low cost through population-based screening approaches that ensure early detection of pre-cancerous lesions, followed by medical referral, timely diagnosis, safe treatment and follow-up.^5^

Co-infection with human immunodeficiency virus (HIV) in the sub-Saharan African countries increases the burden of cervical abnormalities because hr-HPV infections are more prevalent and persistent among HIV-positive women.^6^ A significant number of HIV-positive women are now accessing life-prolonging antiretroviral therapy (ART); therefore, there are more high-risk women living longer at an increased risk of developing ICC in developing countries.^6^

About 80% of cervical cancers in developing countries like Swaziland are diagnosed at an advanced stage, resulting in poor prognosis.^7^ The challenge in these countries is to implement and maintain adequate cervical screening with very limited resources (e.g. infrastructure and health professionals).

The use of Papanicolaou (Pap) smears for early detection of cervical cancer over the past 50 years has led to more than a 70% decrease in the mortality rates in high-income countries, compared to a 19% decrease in low-income countries.^8^ In 1983, Pap was implemented at the Swaziland Central Public Health Laboratory, which remains the only cytology laboratory in the country. This laboratory receives cervical smears of mainly symptomatic women attending health institutions from all over the country. Screening was offered only to a small proportion of women in the two main cities of the country: Mbabane and Manzini. In a report of the status of cytology in Swaziland, 5113 of 11 701 (44%) Pap smears, adequate for diagnosis, received at the Central Public Health Laboratory between 2004 and 2006 were abnormal.^9^ The prevalence of cytological high-grade squamous intraepithelial lesion (HSIL) was 12% (602 of 5113), more than five times the usually reported 1% – 2% in asymptomatic screening populations.^9,10^ Since then, many international studies and programmes have provided evidence of the feasibility and cost-effectiveness of screen-and-treat approaches for cervical cancer prevention in countries with limited resources and difficult scenarios.^11,12^

Visual inspection of the cervix after the application of acetic acid (VIA) is a more attractive and feasible screening strategy for cervical cancer precursors detection than cytology in low-income countries where high-quality cytology-based programmes are difficult to establish or maintain.^13^ Visual inspection with acetic acid is not expensive, can be performed by non-medical health workers and its results are available immediately, allowing treatment of screened positives the same day. In 2009, to fast track the early detection of cervical lesions and facilitate the extension of cervical cancer prevention services in both urban and rural areas, the Swaziland Ministry of Health incorporated the use of VIA followed by treatment with cryotherapy in the national cervical cancer prevention programme.

## Cervical cancer screening and management programme in Swaziland

In 1983, the government of the Kingdom of Swaziland (Ministry of Health) introduced the Pap smear for cervical cancer screening at the National Referral Laboratory, which remains the only functional cytology laboratory in the country. This laboratory receives cervical smears of mainly symptomatic women attending health institutions from all over the country.

However, many international researches and programmes have presented evidence of the feasibility and cost-effectiveness of ‘screen-and-treat’ approaches for cervical cancer prevention in countries of scarce resources. Visual inspection of the cervix after the application of acetic acid is a more attractive and feasible screening approach for cervical cancer precursors detection than cytology in developing countries where high-quality cytology-based programmes are hard to institute or sustain.^13^ Visual inspection with acetic acid is so cheap and user-friendly, can be achieved by any trained nurse and its results are available immediately, allowing treatment of screened positives the same day. In 2009, to quicken the early detection of cervical lesions and facilitate the extension of cervical cancer prevention services across four political regions, the Swaziland Ministry of Health incorporated the use of VIA, followed by treatment with cryotherapy to the national cervical cancer prevention programme. Other treatment methods used include loop electrosurgical excision procedure (LEEP), which is the treatment of choice when a lesion is too large for the cryoprobe or involves the endo-cervical canal or when a histological specimen is needed.^14^ For cervical cancer treatment, according to the national cervical cancer guidelines, patients may be diagnosed by clinicians at clinic level or health centre level and then referred to a regional hospital, which subsequently refers to a National Referral Hospital for further investigations.^14^ However, most of the necessary tests, staging equipment and treatments are not available in Swaziland. Therefore, almost all cervical cancer patients are transferred and treated in South Africa, so a heavey economic burden is experienced by the country.

Therefore, we conducted a retrospective study to describe the distribution and trends of VIA-detected cervical abnormalities over four years in two hospitals that pioneered the implementation of the VIA programme in Swaziland.

## Materials and methods

Records of cervical screening using VIA at the Mbabane Government Hospital and the Realign Fitkin Memorial (RFM) hospital between 2011 and 2014 were retrieved. Visual inspection with acetic acid visits are recorded on designed record books. Nurses performing VIA registered their results themselves and transfer the data to an Excel database with some clerical support.

Following the Ministry of Health guidelines,^14^ VIA was reported as: (1) negative if no acetowhite change is observed, (2) positive if an acetowhite area is noticed in the transformation zone and (3) suspicious for invasive cancer if a growth or ulcerative lesion is observed.

Visual inspection with acetic acid records include the date and result of screening, as well as the age and area of residence of women screened at both centres. No histology data were available from either centre. Age was categorised into five groups: 15–19, 20–29, 30–39, 40–49 and 50–64, and records with ages less than 15 and over 64 years were excluded from the analyses. As the number of screened women younger than 20 years was small, this group was merged with those 20–29 years, or age was split into two groups, less than 30 years and 30–64 years, for certain analyses.

In addition, information on Pap smears and the HIV status from women screened at the Mbabane hospital was retrieved from hospital clinical records whenever available. Papanicolaou results were classified following the Bethesda system^15^ and further grouped into four (or three) categories for analyses: negative, atypical typical squamous cells of undetermined significance (ASCUS) and/or atypical glandular cells of undetermined significance (AGUS), low-grade squamous intraepithelial lesion (LSIL) (or ASCUS and/or LSIL) and HSIL or worse (HSIL+) for statistical analysis. Papanicolaou HSIL was considered a proxy of high-grade cervical disease and was used to calculate positive and negative predictive values of VIA.

HIV status collected from clinical data could be based on recorded ART prescriptions or could be self-reported at the time of screening. None of these data were available for the RFM hospital.

Visual inspection with acetic acid positivity included positive and suspicious for invasive cancer results. Positivity rates (PRs) of VIA with 95% confidence intervals (95% CI) were calculated and used as proxies of cervical abnormalities, overall, by age, by Pap results, by screening centre and by year and month of screening. Visual inspection with acetic acid PRs by these characteristics was compared using the *t*-test for proportions, or the score test for trends as appropriate.

The prevalence of HIV (with 95% CI) was estimated overall by VIA result and age. Odds ratios of HIV status for VIA result (positive vs. negative) were estimated overall and by age using logistic regressions. Potential interactions were evaluated using the likelihood ratio test (LRT). Statistical analysis was carried out using Stata 14.0 SE.

## Ethical consideration

This study was approved by the Scientific Ethics Committee of Swaziland (SEC) (reference no.: MH599C/FW00015267/IRB0009688) and the Biomedical Research Ethics Committee of the University of KwaZulu-Natal (BREC) (reference no.: BE 242/14). As the study was a retrospective study, consent to participate was not applicable.

## Results

**FIGURE 1:**
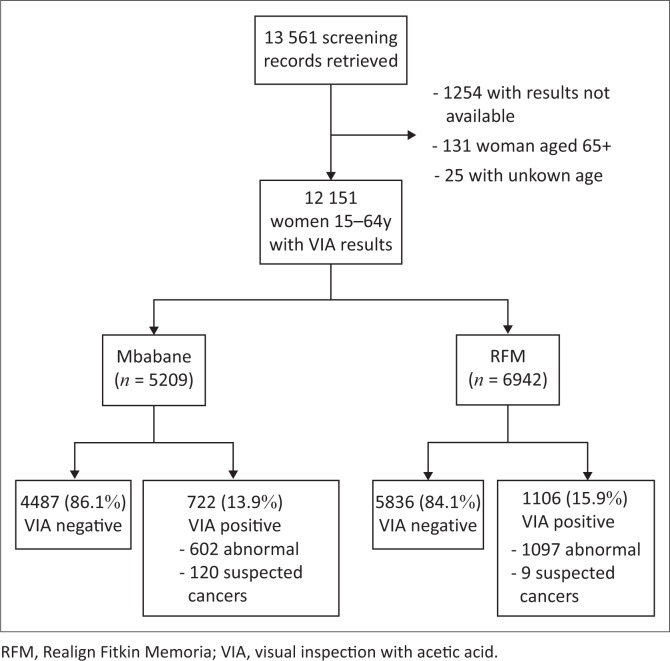
Total number of records of women who attended cervical screening from 2011 to 2014 at Mbabane Government Hospital and Realign Fitkin Memorial hospital, Swaziland.

A total of 13 561 records of women who attended cervical screening from January 2011 to October 2014 at the Mbabane Government Hospital and up to June 2014 at the RFM hospital were reviewed. Records without conclusive VIA results (*n* = 1254) and those from women outside the age range of 15–64 years (*n* = 156) were excluded (Figure 1).

Table 1 shows the main characteristics of women screened with VIA, overall, and separately by VIA abnormal results: positive or suspected cancer. Of the 12 151 VIA exams considered for analysis, 76% were performed in women aged 20–39 years, 60% in women from the Manzini region, 32% in those from Hhohho, 6% in women from Lubombo and 2% in those from Shiselweni. The number of exams per month at Mbabane over 2011 was very small, while at RFM, more than 100 VIAs were performed monthly from March 2011 onwards; a larger proportion (37%) of VIA exams were conducted in 2013 than in other years.

**TABLE 1:** Number of visual inspections with acetic acid (VIA) exams conducted, and percentages of women with VIA-positive results by different characteristics.

Characteristics	No. of VIA exams	%	VIA abnormal results			
			VIA positive		Suspected cancer	
			No.	%	No.	(%)
**Age**						
15–19	403	3.3	35	8.9	1	0.3
20–29	4866	40.0	710	14.6	13	0.3
30–39	4351	35.8	710	16.3	40	1.1
40–49	1829	15.1	202	11.0	31	1.8
50–64	702	5.8	42	6.0	35	5.1
**Region**†						
Hhohho	3884	32.0	462	11.9	63	1.6
Lubombo	710	5.8	134	18.9	10	1.4
Manzini	7301	30.1	1067	14.6	50	0.7
Shiselweni	238	2.0	35	14.7	4	1.7
**Hospital**						
Mbabane	5209	42.9	602	11.6	120	2.3
RFM	6942	57.1	1097	15.8	9	0.1
**Year of screening**						
2011	1951	16.1	422	21.6	17	0.9
2012	3235	26.6	472	14.6	33	1.0
2013	4437	36.5	596	13.4	44	1.0
2014	2528	20.8	209	8.3	35	1.4
**Total**	**12 151**	**-**	**1699**	**14.0**	**129**	**1.1**

RFM, Realign Fitkin Memorial; VIA, visual inspection with acetic acid.

† , Data on region of residence were not available for 18 women in the Mbabane hospital.

Table 2 shows the VIA PR overall by hospital and by age. The overall VIA PR was 15% (1828 of 12 151 women aged 15–64 years) with 1.1% of the exams classified as suspicious for cancer. The overall VIA PR at RFM was 2% higher than that of Mbabane (15.9%, 95% CI: 15.1%–16.8% at RFM, 13.9%, 95% CI: 12.9–14.8 at Mbabane, *p* = 0.02); and this was mainly because of higher rates in women aged 30 and above (17.5%, 95% CI: 16.4–19.0 at RFM vs. 13.4%, 95% CI: 12.3–14.6 for those aged 30–64 at Mbabane, *p* < 0.001). No significant overall trend of VIA PRs by age was observed (*p* for trend=0.15). Visual inspection with acetic acid PRs were about 15% in women less than 40 and then decreased to 9% in those 50–64 years attending the Mbabane hospital, while VIA PRs at RFM overall increased from 8% in women aged 15–19 years to 18% again in those 50 years and older.

**TABLE 2:** Visual inspection with acetic acid results by age and screening centre, 2011–2014.

Screening centre	VIA result†	Age group										All ages	
		15–19	%	20–29	%	30‒39	%	40–49	%	50–64	%	*n*	%
All	Negative	367	91.1	4142	85.1	3595	82.6	1595	87.2	624	88.9	10 323	85.0
	Positive	36	8.9	724	14.9	756	17.4	234	12.8	78	11.1	1828	15.0
Mbabane	Negative	47	85.5	1435	85.3	1600	84.2	903	88.9	502	90.6	4487	86.1
	Positive	8	14.5	248	14.7	301	15.8	113	11.1	52	9.4	722	13.9
RFM	Negative	320	92.0	2707	85.1	1995	81.4	692	85.1	122	82.4	5836	84.1
	Positive	28	8.0	476	14.9	455	18.6	121	14.9	26	17.6	1106	15.9

VIA, visual inspection with acetic acid; RFM, Realign Fitkin Memorial

† , VIA positive includes positive and suspicious for invasive cancer results.

**FIGURE 2:**
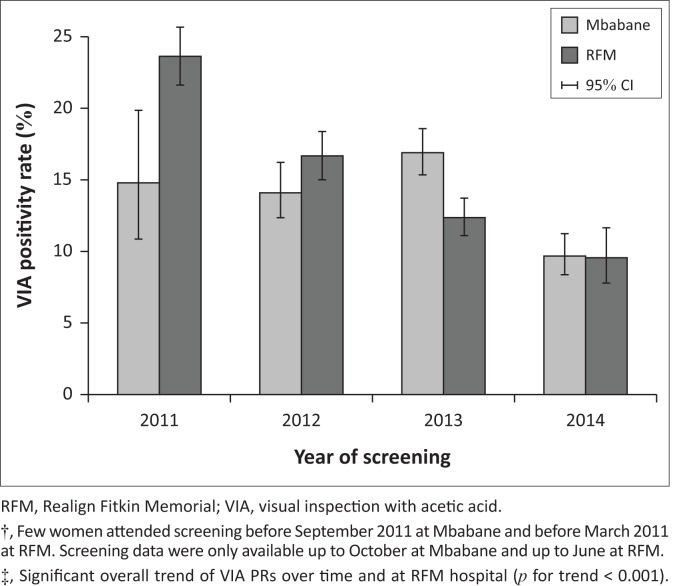
Visual inspection with acetic acid positivity rates (95% confidence interval) over time by hospital.†‡

Figure 2 shows the distribution of VIA PRs over time by hospital, while VIA PRs at RFM decreased clearly over time from 23.6% in 2011 to 9.5% in 2014, such a trend was not evident in exams conducted at Mbabane. However, after excluding months when less than 100 VIAs were performed, the PRs at Mbabane decreased from 14.8% in 2011 to 9.7% in 2014.

There were 3327 Pap smears with results at Mbabane over the four years; 630 (18.9%) were unsatisfactory and among 2697 satisfactory smears, 84% of them were negative, 251 (9%) were ASCUS and/or LSIL and 181 (7%) were HSIL or worse (HSIL+). Visual inspection with acetic acid was positive in only 182 (8%) women with negative Pap smears, but 11 (6% of 182) exams were considered suspicious for cancer. Among 432 women with abnormal cytology, 34% (149) were VIA positive; in particular, of 181 women with HSIL, 67 (37%) were positive on VIA, and 15 (8%) were considered suspected cancers. The percentage of Pap HSIL increased with age from 5% in women less than 30 years to 10% in women less than 50 years and older and a similar trend was observed for VIA classified as suspicious for cancers which increased from 0.8% in the youngest age group to 6% in the older one (Table 3).

**TABLE 3:** Papanicolaou results and human immunodeficiency virus status by visual inspection with acetic acid result and age at Mbabane, 2011–2014.

Variables	VIA results						Pap adequate results						HIV available status			
	All (*N* = 5209)		VIA Positive		VIA SC		All (*N* = 2697)		Pap ASCUS+		Pap HSIL+		All (*N* = 4578)		HIV-positive	
	No.	%†	No.	%‡	No.	%‡	No.	%†	No.	%‡	No.	%‡	No.	%†	No.	%‡
**VIA**																
Negative	4487	-	-	-	-	-	2366	87.7	283	12.0	114	4.8	3935	85.9	1300	33
Positive	602	-	-	-	-	-	302	11.2	131	43.4	52	17.2	539	11.8	334	62
Suspected cancer	120	-	-	-	-	-	29	1.1	18	62.1	15	51.7	104	2.3	68	65.4
**Pap**																
Negative	2265	84	182	8.0	11	0.5	-	-	-	-	-	-	2099	68.2	676	32.2
ASCUS and/or LSIL	251	9.3	82	32.7	3	1.2	-	-	-	-	-	-	232	7.5	149	64.2
HSIL	181	6.7	67	37.0	15	8.3	-	-	-	-	-	-	178	5.8	94	52.8
Inadequate	630	-	124	19.7	36	5.7	-	-	-	-	-	-	569	18.5	235	36.5
Not available	1882	-	267	14.2	55	2.9	-	-	-	-	-	-	1500	-	548	36.5
**HIV status**																
Negative	2876	62.8	241	8.4	36	1.3	1590	63.4	167	10.5	84	5.3	-	-	-	-
Positive	1702	37.2	402	23.6	68	4.0	919	34.6	243	26.4	94	10.2	-	-	-	-
Not available	631	-	79	12.5	16	2.5	188	-	22	11.7	3	1.6	-	-	-	-
**Age**																
15–29	1738	33.4	256	14.7	14	0.8	983	36.5	140	14.2	51	5.1	1574	34.4	534	33.9
30–39	1901	36.5	301	15.8	40	21.1	955	35.4	160	16.8	64	6.7	1678	36.7	760	45.3
40–49	1016	19.5	113	11.1	31	3.0	535	19.8	92	17.2	43	8.0	858	18.7	300	35.0
50–64	554	10.6	52	9.4	35	6.3	224	8.3	40	17.9	23	10.3	468	10.2	108	23.1

Note: All include women with corresponding available results. VIA+ includes positive and suspicious for invasive cancer results. PAP ASCUS+ includes paps reported as ASCUS, LSIL and HSIL and cancer. Pap HSIL includes only paps reported as HSIL or cancer.

VIA, visual inspection with acetic acid; Pap, Papanicolaou.

† , Percentages of those with a particular test result (or age group) = No. with particular result/(No. in ‘ALL’ – ‘Inadequate and/or Not available’ test results).

‡ , Percentage of those in row who were positive on each test (or age group).

Of 4578 women with known HIV status, 1702 (37.2%, 95% CI: 35.8–38.6) were HIV-positive; VIA was positive in 8% of HIV-negative women and in 24% of HIV-positive women. The highest HIV prevalence was observed among women of 30–39 years (45%), HIV prevalence decreased afterwards, independently of VIA result (Supplementary Figure 3).

**FIGURE 3:**
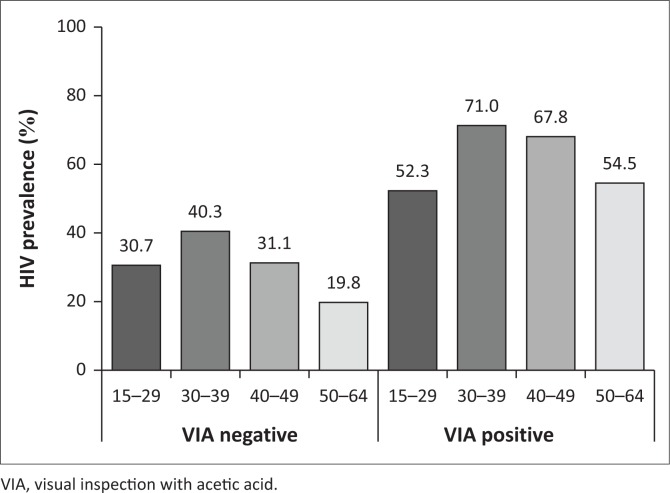
Human immunodeficiency virus prevalence by age and visual inspection with acetic acid results at Mbabane, 2011–2014

LSIL, low-grade squamous intraepithelial lesion; HSIL, high-grade squamous intraepithelial lesion; Pap, Papanicolaou; VIA, visual inspection with acetic acid; HIV, human immunodeficiency virus.

There were 2509 women with adequate Pap smears and known HIV status, 410 (10%) of them had abnormal cytology (232 ASCUS and/or LSIL and 178 HSIL) and 243 (59% of 410) were HIV-positive.

In the absence of histology results, assuming PAP HSIL+ as a proxy of high-grade cervical disease and therefore as gold standard, the sensitivity and specificity of VIA to detect Pap HSIL were 37% (95% CI: 30.0–44.5) and 89.5% (95% CI: 88.2–90.7), while the positive and negative predictive values of VIA were 20.2% (95% CI: 16.0–25.0) and 95.2% (95% CI: 94.2–96.0).

In a multivariate logistic model including age, cytology abnormalities (Pap ASCUS+) and HIV positivity, after adjusting for the other two factors, HIV-positive women were 2.9 times more likely to have a positive VIA (OR: 2.9, 95% CI: 2.3–3.8) than HIV-negative women, similarly, compared to women with normal Pap smears; those with abnormal cytology were 4.8 times more likely to be VIA-positive (Table 4).

**TABLE 4:** Odds ratios (95% CI) relating human immunodeficiency virus status and Pap results to visual inspection with acetic acid positivity.

Results	No. of women	No. of VIA+	VIA+ (%)	UOR	95% CI	AOR	95% CI
HIV-negative	2876	241	8.4	1.0	-	1.0	-
HIV-positive	1702	402	23.6	**3.4i**	**2.8–4.0**	**2.9**	**2.3–3.8**
Pap negative	2265	182	8.0	1.0	-	1.0	-
Pap ASCUS+	432	149	34.5	**6.0j**	**4.7–7.7**	**4.8**	**3.7–6.3**

VIA+, VIA positive including positive and suspicious for invasive cancer; No., number; UOR, unadjusted odds ratio; AOR, odds ratio adjusted for HIV status; Pap ASCUS+, result and age, model based on 2509 observations with complete data; VIA, visual inspection with acetic acid; Pap, Papanicolaou.

† , HIV-positive women significantly associated with VIA-positive;

‡ , Pap ASCUS+ women significantly associated with VIA-positive.

## Discussion

In this retrospective study, we summarised the distribution of cervical abnormalities based on VIA results among 13 561 women who attended cervical screening in two main hospitals of Swaziland from 2011 to 2014, following the implementation of VIA in 2009 as part of the national cervical screening programme. Despite not having data for some months in 2014, it was observed that after the introduction of VIA in each hospital, the number of VIA exams increased annually from 1951 in 2011 to 4437 in 2013, when the largest proportion of VIA exams were conducted (38.8% at Mbabane, 34.8% at RFM).

Our study showed an overall VIA PR of 15% (95% CI: 14.4–15.7) among women aged 15–64 years with 1.1% of VIA exams classified as suspicious for cervical cancer. In addition, there was a much smaller proportion of VIA classified as suspected cancer in RFM than at Mbabane (0.1% *v* 2.3%). These differences could be explained by differences in the criteria used to classify exams and by observer variability. Visual inspection with acetic acid exams were conducted by one single nurse in each centre, both of them were trained at the same time by experts in Zambia; therefore, it is unlikely that differences are because of VIA positivity definition. In addition, there are potential issues of overcall versus under-call between the two nurses. It is known that VIA PRs vary largely compared to other screening test results and that VIA heavily depends on human judgement.^16^ As only one nurse per centre performed VIA, it is likely that the differences represent observer variability. It has been proposed that this variability can be lessened with the establishment of VIA proficiency schemes that can provide certification to VIA providers. Unfortunately, in poor-resource settings, the availability of training and supervision may be limited, and other options to ensure quality of VIA should be explored. Nevertheless, overall observed VIA PRs are within limits of previous studies.^17,18,19^

The prevalence of HIV was 37.2% among 4578 women aged 15–64 years with known HIV status (self-reported or based on clinical records) attending the Mbabane hospital. Even though HIV status was only available for one hospital, the results are likely to be representative of all women in Swaziland, as they are similar to those reported by the 2011 Swaziland HIV Incidence Measurement Survey (SHIMS), in which the HIV prevalence in women aged 18–49 years was 39%.^20^

It is clear that cervical abnormalities are more prevalent among HIV-positive women than among HIV-negative women. A review of studies published before 2007 that evaluated the association between HIV infection, HPV infection and cervical neoplasia, and the impact of using highly active ART (HAART) on the association, reported ratios of the prevalence of HPV infection, LSIL and HSIL in HIV-positives compared to HIV-negatives.^21^ Among women who participated in 11 studies in Africa, prevalence ratios ranged between 1.0 and 3.6 for HPV, between 1.6 and 8.8 for LSIL and 3.1 and 5.1 for HSIL; thus, prevalence ratios increased by grade of abnormality, confirming that HIV infection is strongly associated with higher HPV-persistent infection, which leads to higher prevalence of squamous intraepithelial lesions, and eventually to higher rates of ICC.^21^ However, the evidence was not that clear for HAART use, and although it seems that HAART has little or no beneficial effect on the natural history of cervical lesions in HIV-positive women, its use increases their life expectancy.^21^ In addition, as life expectancy increases, so does the likelihood of developing cervical cancer if precancer lesions are not detected and treated on time. In low- and middle-income countries, organised cervical screening does not exist or has usually been unsuccessful. Even when there is enough infrastructure and human resources available, the organisation of screening activities such as inviting women, returning results on time and, most importantly, ensuring that women with cervical lesions are treated, are difficult to achieve. It demanding less complex schemes, such as VIA, followed by cryotherapy (see-and-treat) preferably carried out in one single visit. A recent study conducted in three countries: Guyana in South America, Cote d’Ivoire in West Africa and Tanzania in East Africa, where 34 921 women were screened using VIA in a single-visit approach, HIV-positive women had higher odds of being VIA positive (OR: 1.9, 95% CI 1.8–2.2) and of having large lesions requiring referral (OR: 1.9, 95% CI: 1.5–2.5) than HIV-negative women.^22^ These results, although less strong, are in line with ours as we found that HIV-positive women were over three times more likely to have cervical abnormalities detected at VIA than HIV-negatives (OR: 3.4: 95% CI: 2.8–4.0), and that this HIV effect was stronger among women of 30 years and older (OR: 4.2, 95% CI: 3.3–5.2), than among those younger than 30 (OR: 2.5, 95% CI: 1.9–3.3).

A subset of 2697 records retrieved from the Mbabane hospital contained Pap results. The overall percentage of cytological abnormalities was 16% (95% CI: 14.7–17.5); 3% of them were ASCUS and/or AGUS, 6% LSIL and 7% HSIL or cancer). In a previous report of 12 323 smears read at the Central Public Health Laboratory (CPHL), the proportion of cytological abnormalities was 44%, almost threefold ours^9^; however, while we have retrieved records of mostly asymptomatic women attending screening, most smears read at the CPHL were from symptomatic women. We further cross-tabulated VIA with Pap results, observing that the percentage of VIA positive results increased by cytological grade; from 8% in women with negative cytology, 27% in those with ASCUS and/or AGUS, 35.8% in LSIL and 37% in those with HSIL or cancer. It is important to highlight that although there were only 29 VIAs classified as suspected for cancer with known Pap results, 11 (38%) of them were on women with negative cytology and 15 (52%) in those with HSIL+. Local guidelines indicate that these women should be referred to colposcopy and biopsy and should be treated based on histology results. A number of problems arise with these cases: there are only two pathologists (recently appointed) in the country, very few gynaecologists and no radiotherapy facilities in Swaziland. Invasive cervical cancer cases are reported to a referral scheme called Phalala, whose committee decides whether a cancer case is referred for chemotherapy and radiotherapy to South Africa. This process can take up to four months, and the decision for referral, the treatment given and the clinical status after treatment are generally not reported back to the local doctor. Thus, we do not know the final diagnoses of these women and whether they were treated or not.

To our knowledge, this study represents the first evaluation of VIA results after its introduction in the Swaziland National Cervical Screening programme. The main strength of our study relies on the large number of VIA screens performed in women, residents from all political regions of the country, who attended two major hospitals over four years. However, although the records retrieved, we assumed, were representative of all women in Swaziland, we cannot rule out potential information and reporting bias, as we have used routine-collected data. Therefore, we accept that biases may have been greater because this is an analysis based on routine-collected data, that is, we did not have any control over the data collection. In fact, we were able to retrieve data on Pap smears and HIV-reported status only from a small subset of women. In addition, although we can assume that at least in the two hospitals included in this report adherence to the screen-and-treat protocol was high, and that the majority of VIA positives eligible for cryotherapy were treated, we were unable to retrieve data to confirm this. Another major limitation of our study is the lack of colposcopy and histological confirmation of disease, which was non-existent, as only recently two pathologists have been appointed in the country. We still estimated the performance of VIA using Pap HSIL as gold standard; in this scenario, it is of concern the very low sensitivity of VIA, 37%, highlighting that there may be a number of women with cervical disease left untreated. Such may be because of challenges of implementing screening such as the low number of health service providers and limited training opportunities.

There is overwhelming evidence that human papillomavirus (HPV) DNA testing is more sensitive than cytology and VIA for the detection of high-grade cervical lesions.^23,24^ It has also been demonstrated that a negative HPV test provides long-term protection against cervical neoplasia and stronger reassurance of not developing invasive cancer, and this protection can be translated into fewer screens over life, facilitating adequate screening of vulnerable populations in particular in low- and middle-income countries. Nevertheless, even within HPV-based screening, the major challenges associated with the process of screening mentioned above will remain; in particular, those associated with ensuring that women receive their screening results and are properly evaluated and treated. The difficulties associated with following-up women could be reduced by using HPV testing within screen-and-treat (HPV and cryotherapy) schemes, with or without VIA triage, that should be considered as an alternative to VIA-based screening once a validated HPV test becomes affordable enough to be introduced and scaled up in countries with limited resources.

## Conclusion

In conclusion, based on our findings, cervical abnormalities represent a public health problem that needs to be addressed urgently by strengthening, and expanding the existing cervical cancer screening in the country, with a priority among HIV-positive women. Lobbying and advocating for the development of a cervical cancer screening policy, as well as the introduction of HPV vaccine at national level is needed to implement a comprehensive cervical cancer prevention programme. The results found in this retrospective study call for improved health education programmes on cervical cancer screening and potential-associated risk factors such as sexually transmitted infections, in particular HPV, among high-risk groups. It also calls for the evaluation of alternative screening schemes that include HPV testing that can offer further reassurance to screened negatives of not developing cervical cancer for several years. Nonetheless, population-based screening using low cost VIA, if followed by treatment of VIA positives, is currently the best screening alternative in limited-resourced settings until a validated HPV test becomes affordable.
